# Diagnostic Prediction Models for Primary Care, Based on AI and Electronic Health Records: Systematic Review

**DOI:** 10.2196/62862

**Published:** 2025-08-22

**Authors:** Liesbeth Hunik, Asma Chaabouni, Twan van Laarhoven, Tim C Olde Hartman, Ralph T H Leijenaar, Jochen W L Cals, Annemarie A Uijen, Henk J Schers

**Affiliations:** 1 Department of Primary and Community Care, Research Institute for Medical Innovation, Radboudumc, Geert Grooteplein Zuid 21, Nijmegen, 6525 GA, The Netherlands, 31 243618181; 2 Institute for Computing and Information Science, Radboud University, Nijmegen, The Netherlands; 3 Department of Family Medicine, Care and Public Health Research Institute, Maastricht University, Maastricht, The Netherlands

**Keywords:** primary care, electronic health records, artificial intelligence, EHR, AI, systematic review, decision-making, AI-based diagnostic, applicability, assessment tool

## Abstract

**Background:**

Artificial intelligence (AI)–based diagnostic prediction models could aid primary care (PC) in decision-making for faster and more accurate diagnoses. AI has the potential to transform electronic health records (EHRs) data into valuable diagnostic prediction models. Different prediction models based on EHR have been developed. However, there are currently no systematic reviews that evaluate AI-based diagnostic prediction models for PC using EHR data.

**Objective:**

This study aims to evaluate the content of diagnostic prediction models based on AI and EHRs in PC, including risk of bias and applicability.

**Methods:**

This systematic review was performed according to the PRISMA (Preferred Reporting Items for Systematic Reviews and Meta-Analyses) guidelines. MEDLINE, Embase, Web of Science, and Cochrane were searched. We included observational and intervention studies using AI and PC EHRs and developing or testing a diagnostic prediction model for health conditions. Two independent reviewers (LH and AC) used a standardized data extraction form. Risk of bias and applicability were assessed using PROBAST (Prediction Model Risk of Bias Assessment Tool).

**Results:**

From 10,657 retrieved records, a total of 15 papers were selected. Most EHR papers focused on 1 chronic health care condition (n=11, 73%). From the 15 papers, 13 (87%) described a study that developed a diagnostic prediction model and 2 (13%) described a study that externally validated and tested the model in a PC setting. Studies used a variety of AI techniques. The predictors used to develop the model were all registered in the EHR. We found no papers with a low risk of bias, and high risk of bias was found in 9 (60%) papers. Biases covered an unjustified small sample size, not excluding predictors from the outcome definition, and the inappropriate evaluation of the performance measures. The risk of bias was unclear in 6 papers, as no information was provided on the handling of missing data and no results were reported from the multivariate analysis. Applicability was unclear in 10 (67%) papers, mainly due to lack of clarity in reporting the time interval between outcomes and predictors.

**Conclusions:**

Most AI-based diagnostic prediction models based on EHR data in PC focused on 1 chronic condition. Only 2 papers tested the model in a PC setting. The lack of sufficiently described methods led to a high risk of bias. Our findings highlight that the currently available diagnostic prediction models are not yet ready for clinical implementation in PC.

## Introduction

### Background

The diagnostic process is a core task of general practitioners (GPs). However, making a diagnosis may be a challenging task given the diversity, complexity, and early presentation of symptoms. Clinical prediction models are intended to improve the diagnostic process [1]. These models can support the health care provider by predicting serious illness [2]. In the last years, the interest in artificial intelligence (AI) techniques for the development of prediction models has been growing [3,4]. AI-based prediction models could aid in decision-making for faster and more accurate diagnoses, with more diagnostic efficiency that can benefit patients’ health [5-8]. Examples are prediction tools that can predict colorectal cancer in patients [9,10].

Clinical prediction models used to be built on data from large databases, such as data collected for research purposes, claim data, or data from electronic health records (EHRs) [11,12]. EHR data consist of structured data, which are data in standardized format, and unstructured data, which are free-text data. Primary care (PC) EHR data provide extensive and longitudinal data from a patient’s health trajectory and changes over time. AI might prove to be a valuable method to extract clinically useful and actionable insight from this vast and complex source of patient data [13]. For that reason, AI has the potential to transform EHR data into a valuable tool for predicting diagnosis in daily PC practice.

Reviews on the value of AI in PC are scarce, and previous research had different aims. For example, Kueper et al [14] provided an overview of diagnostic prediction models based on AI in PC. However, the authors did not assess the quality of these diagnostic prediction models. Other research in this field explored AI systems in community-based primary health care [15] or focused on different machine learning (ML)–based diagnostic and prognostic models that predicted a health care condition [16]. As AI has the potential to support and improve the diagnostic process, high-quality and validated prediction models are crucial in order to ensure patient safety after clinical implementation. Although a variety of prediction models for PC have been developed, to our knowledge, there are currently no systematic reviews on AI-based diagnostic prediction models for PC using EHR data.

### Objective

Evaluation of the content and quality assessment of AI-based diagnostic prediction models using EHRs in PC was largely lacking in current literature. Therefore, we systematically reviewed the literature in order to critically evaluate the content of these AI-based diagnostic prediction models, including risk of bias and applicability.

## Methods

### Study Design

We performed a systematic review according to the PRISMA (Preferred Reporting Items for Systematic Reviews and Meta-Analyses) guidelines [17] (the PRISMA checklist is provided in Checklist 1). The protocol for this study was registered in PROSPERO (nr: CRD42022320002). The research team included stakeholders such as practicing GPs, researchers, methodologists, and AI experts in the design, analysis, and reporting of the study.

### Search Strategy and Study Selection

Our search was adapted from the search strategy developed by Kueper et al [14]. It combines two concepts including a wide range of different terms used to describe the concepts: (1) artificial intelligence and (2) primary care (for full search strategy, see Multimedia Appendix 1, part 1 [18-67]). EHRs were not part of the search strategy, because literature suggests that we might miss important studies when including EHRs or related terms in the search strategy [13]. We searched in the following databases: MEDLINE, Embase, Web of Science, and Cochrane. There were no restrictions concerning the publication date. The last search update was conducted on August 28, 2023. We focused on intervention and observational studies. We excluded systematic reviews, meta-analyses, case studies, editorials, protocols, and conference posters or abstracts. Full text had to be available to be selected for screening. The literature had to be written in English or Dutch. Duplicate publications were removed with EndNote 20.

### Inclusion and Exclusion Criteria

Four inclusion criteria were used to select the papers: (1) primary care focus: this included PC data, models that were tested in a PC setting, or PC had to be specifically mentioned in the aim of the study; (2) diagnostic prediction model: models had to predict a health condition applicable during a GP’s consultation; prediction models that identified a disease in a database, rather than predicting a disease for an individual, were excluded; (3) AI: this included all ML and deep learning techniques; we directed our focus to data-driven prediction models without using medical images as input data; and (4) EHR-based data: EHR data had to be used for the development or validation of the model. EHRs were defined as PC data from EHRs, medical records, or clinical notes. See Multimedia Appendix 1, part 2 [18-67], for the full screening guidance.

Title and abstract screening was done in management software Rayyan (rayyan.ai) by 2 independent reviewers (LH and LvdH). Conflicts were resolved by a third reviewer (AU). Full-text screening was done by the same independent reviewers. Conflicts were resolved by discussion, and if no consensus was reached, they were resolved by a third reviewer (AU). Backward citation searching was conducted on the included papers and finished on November 7, 2023.

### Data Extraction and Quality Assessment

Data extraction of included papers was done by 2 independent reviewers (LH and AC). They used a standardized data extraction form adapted from the Checklist for Critical Appraisal and Data Extraction for Systematic Reviews of Prediction Modelling Studies (CHARMS) [68]. Basic information was extracted from all papers. The extraction of more detailed information was focused on EHR-based papers. For all papers (EHR and non-EHR papers), we extracted general information (first author, year of publication, title, data source, and country of data source), study design (retrospective or prospective), and outcome (predicted health condition). For the EHR papers, we additionally extracted dataset information (name of the dataset and sample size: number of participants used for model training, testing, or validation), AI technique, and predictors (the potentially used predictors used to develop the model). Risk of bias and applicability were assessed using PROBAST (Prediction Model Risk of Bias Assessment Tool). This tool includes 20 signaling questions divided into 4 domains (participants, predictors, outcome, and analysis) [69,70]. Overall judgment (ie, low, unclear, or high) of risk of bias is based on the 4 domains. If 1 domain is considered to have a high risk of bias, the overall judgment is scored as a high risk of bias. If at least 1 domain is considered to have an unclear risk of bias (without a domain with high risk of bias), the overall judgment is scored as unclear risk of bias. Applicability concern was rated based on 3 domains (participants, predictors, and outcome) and an overall judgment of applicability (ie, low, unclear, or high) was also given with the same approach as the risk-of-bias scoring. Applicability evaluation depends on the review question [69], and we translated applicability assessment as usability of the diagnostic prediction model in a PC setting. Conflicts in data extraction between the 2 reviewers (LH and AC) were resolved by discussion, and if no consensus was reached, they were resolved by a third reviewer (TvL).

## Results

### Description of Included Studies

**Figure 1.**
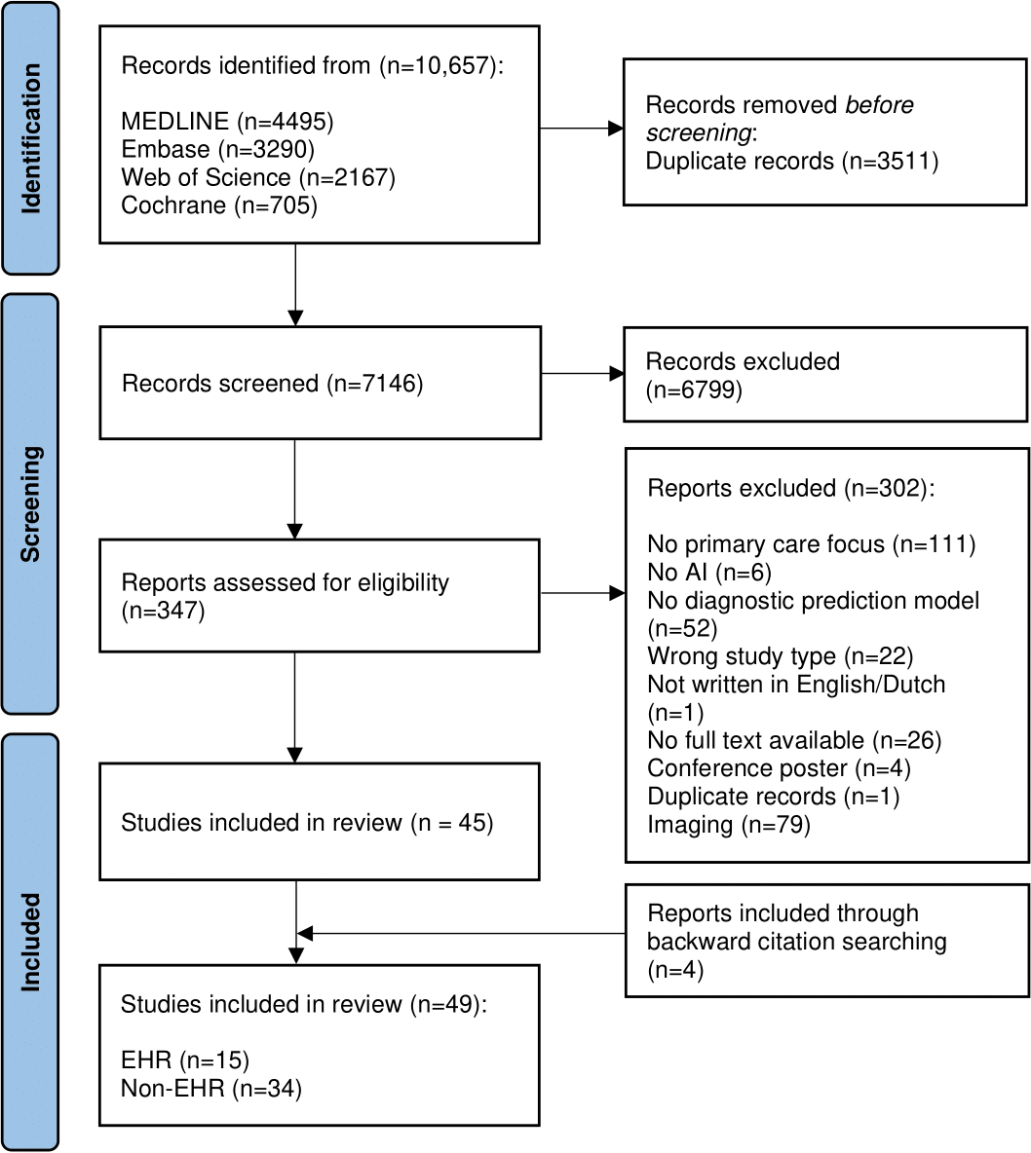
PRISMA (Preferred Reporting Items for Systematic Reviews and Meta-Analyses) flowchart of study selection. AI: artificial intelligence; EHR: electronic health record.

We retrieved 10,657 records using our search strategy. After duplicate removal, we conducted title and abstract screening on 7146 records. A total of 347 records met the eligibility criteria for full-text screening. After full-text screening, 45 records were included. Backward citation searching yielded an additional 4 papers. A total of 49 papers were thus included in the review (Figure 1). Of the included papers, we identified 15 EHR papers and 34 non-EHR papers. A detailed description of the 34 non-EHR papers can be found in Multimedia Appendix 1, part 3 [18-67]. The data used in these 34 papers were collected from different sources, including secondary care datasets (n=17), questionnaires (n=4), and the knowledge of different health care providers (n=5).

### Overview of the EHR-Based Papers

Of the 15 EHR papers, 13 (87%) included the development of a prediction model [18-30]. In Table 1, the data extraction per paper can be found. The included EHR papers covered various outcomes, mostly chronic conditions (11/15, 73%) [19-28,31]. The most frequent predicted outcomes were dementia (3/15, 20%) [19,20,23], asthma (3/15, 20%), or chronic obstructive pulmonary disease (COPD) (3/15, 20%) [21,26,31]. Other study outcomes are shown in Table 1. All included papers used predictors registered in EHRs. Predictors included findings from clinical examination (n=6) [19,25,26,28,31,32], laboratory results (n=5) [21,22,25,28,32], and medication (n=4) [19,21,24,29]. All models used structured data.

Two papers externally validated and tested a prediction model in a PC setting [31,32]. One paper had a prospective approach and tested the diagnostic performance of a prediction model for asthma and COPD [31]. Ten papers (10/15, 67%) were published after 2020 [18,21-24,26,27,29,31,32]. Most data sources used in the studies originated from Europe (8/15, 53%) [18-22,24,27,31], followed by North America (5/15, 33%) [23,25,28,29,31,32] and Asia (2/15, 13%) [26,30].

**Table 1.** Extracted information from electronic health record papers.

Author, year	Country	Study type	Study design	Outcome	Dataset	Participants and inclusion criteria	Predictors	AI technique
Barnes et al (2020) [23][Bibr R26]	United States	Developmental	Retrospective cohort study	To identify patients at high risk of unrecognized dementia	Data from Kaiser Permanente Washington	4330 participants aged at least 65 years, community member with no dementia	Demographics, diagnosis, vital signs, health care usage, medication	LR^a^
Briggs et al (2022) [22][Bibr R25]	United Kingdom	Developmental	Nested case-control study	To predict risk of esophagogastric cancer	Data from General Practice Research Database	40,348 participants with esophagus or gastric cancer (7471 cases and 32,877 matched controls) diagnosed after 2000 (aged ≥40 years)	Demographics, symptoms, laboratory results	RF^b^, SVM^c^, LR, NB^d^, XGBoost^e^
Dhanda et al (2023) [32][Bibr R35]	United States	Developmental + Validation	Retrospective cohort study	To predict urine culture result without microscopy data to predict urinary tract infection	Data from emergency department (developmental phase). Data from primary care outpatient family medicine department at University of Kansas Medical Center (external validation)	80,859 participants (80,387 development, 472 external validation) with an ordered urinalysis and urine culture	Demographics, urine analysis, vital signs, symptoms, history of urinary tract infection, higher risk of clinical features	XGBoost, RF, NN^f^
Dros et al (2022) [Bibr R27][24]	Netherlands	Developmental	Nested case-control study	To identify primary Sjögren syndrome	Data from Nivel Primary Care Database linked with Diagnosis Related Groups Information System dataset	930,590 participants (1411 cases, 1411 controls for training phase and all of the 929,179 controls for testing phase), with primary Sjögren syndrome from 2017	Demographics, diagnosis, medication, health usage	LR, RF
Ellertsson et al (2021) [18][Bibr R21]	Iceland	Developmental	Retrospective cohort study	To diagnose common clinical headaches (cluster headache, migraine [with or without aura], tension headache)	Data from 15 primary Health Care of the Capital Area clinics	Unknown number of participants, 800 clinical notes from patients with 4 headache diagnoses from 2006 to 2020	Headache symptoms, sex, age, family history	RF
Ford et al (2019) [19][Bibr R22]	United Kingdom	Developmental	Nested case-control study	To detect dementia	Data from Clinical Practice Research Datalink data	93,120 participants (46,560 cases with a dementia diagnosis code between 2000 and 2012, 46,560 controls)	Symptoms of physical or cognitive frailty, medical history, health care usage, ethnicity, family history of dementia, intoxications, BMI, blood pressure, psychological diagnoses, and treatment	RF, NB, SVM, NN
Jammeh et al (2018) [20][Bibr R23]	United Kingdom	Developmental	Case-control study	To identify undiagnosed dementia	NHS Devon dataset with 18 participating GP^g^ surgeries	3063 participants (850 cases with a dementia diagnosis code, 2213 controls)	Demographics, long-term conditions, and consultations	LR, RF, NB, SVM
Kocks et al (2023) [31][Bibr R34]	Netherlands	Validation	Prospective observational study	To diagnose asthma, COPD^h^, or asthma-COPD overlap	Data from Nivel Primary Care Database	116 cases, tested on 180 specialists from 9 countries (external validation) from patients aged ≥40 years, with complete data file	Symptoms, BMI, spirometry scores, smoking, diagnosis of chronic or allergic rhinitis, age at onset of respiratory disease	Multinomial LR
LaFreniere et al (2016) [25][Bibr R28]	Canada	Developmental	Case-control study, nested is unclear	To predict hypertension	EHR data from Canadian Primary Care Sentinel Surveillance Network	379,027 participants (185,371 cases with hypertension, 193,656 controls with no hypertension and with no 8 specific chronic conditions)	Demographics, BMI, blood pressure, laboratory results	NN
Lin et al (2023) [26][Bibr R29]	China	Developmental	Retrospective cohort study	To identify COPD	Public health data from EHRs and electronic medical records of Chinese residents	1875 participants with lung symptoms or chronic lung disease	Demographics, smoking, BMI, chronic cough, shortness of breath, biofuel use, and family history. Based on the questionnaire for COPD	18 methods, including: Decision tree, LR, discriminant analysis (linear and quadratic), SVM, gradient boosting classifiers, NN, Gaussian process classifier, KNN^i^, NB
Mariani et al (2021) [21][Bibr R24]	Netherlands	Developmental	Retrospective cohort study	To diagnose asthma and COPD or asthma-COPD overlap	Data from Dutch primary care laboratory in Groningen	3659 participants with asthma or COPD from 2007 to 2017	Demographics, symptoms, diagnosis, medication, laboratory results, referrals, spirometry results	SVM, RF, KNN
Nemlander et al (2023) [27][Bibr R30]	Sweden	Developmental	Nested case-control study	To identify nonmetastatic colorectal cancer	Regional administrative health care database from Västra Götaland Region	2681 participants (542 cases with a cancer diagnosis, 2139 controls)	Nonmetastatic colorectal cancer stage, number of GP consultations, diagnosis codes	Stochastic gradient boosting
Perveen et al (2016) [28][Bibr R31]	Canada	Developmental	Retrospective cohort study	To classify diabetes mellitus in 3 adult age groups	EHR data from Canadian Primary Care Sentinel Surveillance Network	4678 participants (377 cases of diabetes, 4301 controls with no diabetes) with all documented risk factors	Demographics blood pressure, laboratory results	Decision tree, bagging, ADAboost
Singh et al (2022) [29][Bibr R32]	United States	Developmental	Retrospective cohort study	To predict anterior segment vision-threatening disease (asVTD)	EHRs of the University of Michigan	2942 participants with anterior segment eye complaint (133 cases with asVTD, 2809 controls) with PC notes with ophthalmologist visit	Demographics, history of eye problems, symptoms, medication	Elastic net LR
Su et al (2019) [30][Bibr R33]	China	Developmental	Retrospective cohort study	Top 100 diagnoses (within general diagnoses)	National Hospital Ambulatory Medical Care Survey and the National Ambulatory Medical Care Survey	Unknown number of participants, top 100 diagnosis selected from 2,000,000 records	Demographics, symptoms, past medical history	NN

a LR: logistic regression.

b RF: random forest.

c SVM: support vector machine.

d NB: naïve Bayes.

e XGBoost: extreme gradient boosting.

f NN: neural network.

g GP: general practitioner.

h COPD: chronic obstructive pulmonary disease.

i KNN: K-nearest neighbors.

### AI Technique

All of the included studies performed at least 1 supervised AI technique (Table 1). The most used AI techniques were random forest (9 papers), logistic regression (7 papers), support vector machines (5 papers), boosting algorithms (5 papers), neural networks (5 papers), and naïve Bayes (4 papers).

### Quality Assessment: Risk of Bias

None of the studies assessed by the PROBAST tool had a low risk of bias. We found a high risk of bias in 9 studies (9/15, 60%) and an unclear risk of bias in 6 studies (6/15, 40%; Table 2). In Multimedia Appendix 1, part 4 [18-67], the full assessment of the PROBAST tool can be found.

**Table 2.** Risk of bias per domain using the Prediction model Risk Of Bias ASsessment Tool.

	Participants	Predictors	Outcome	Analysis	Overall
Barnes [23]	Low	Low	Low	Unclear	Unclear
Briggs et al [22]	Low	Unclear	Unclear	Unclear	Unclear
Dhanda et al [32]	High	Low	Unclear	Unclear	High
Dros et al [24]	Low	Low	Unclear	High	High
Ellertsson et al [18]	Low	Unclear	Low	High	High
Ford et al [19]	Unclear	Low	Low	Unclear	Unclear
Jammeh et al [20]	High	Unclear	Unclear	Unclear	High
Kocks et al [31]	Low	Low	High	High	High
LaFreniere et al [25]	Unclear	Low	Unclear	Unclear	Unclear
Lin et al [26]	Unclear	Low	Unclear	High	High
Mariani et al [21]	Unclear	Low	Unclear	Unclear	Unclear
Nemlander et al [27]	High	Low	Unclear	Unclear	High
Perveen et al [28]	Low	Unclear	High	High	High
Singh et al [29]	Low	Low	Unclear	High	High
Su et al [30]	Unclear	Unclear	Unclear	Unclear	Unclear

The most significant source of bias was found in the analysis domain. The main reasons for the high risk of bias in this domain were the insufficient number of participants with the outcome (5/15, 33%) [18,24,26,29,31] and irrelevant model performance measures that were used to evaluate the model (2/15, 13%) [28,31]. The main reasons for an unclear risk of bias in the analysis domain were lack of clarity on how missing data were handled (10/15, 67%) [18-20,22,23,27-30,32], and on how the predictors and their assigned weights in the final model correspond to results from the reported multivariate analysis (9/15, 60%) [18,20,21,25-30]. Although measures of calibration are not part of the signaling questions of the PROBAST, we noticed that only 4 papers (4/15, 27%) [22,23,29,32] used calibration to assess the performance of the model.

The second significant source of bias was found in the outcome domain. The main reasons for the high risk of bias in this domain were the determination of the predictors with a prior knowledge of the outcome (1/15, 7%) [31] and not excluding the predictors from the outcome definition (2/15, 13%). For example, Perveen et al [28] included fasting glucose levels to predict diabetes and Kocks et al [31] included spirometry findings to predict asthma and COPD. The 2 main reasons for an unclear risk of bias in the outcome domain were lack of clarity on the time interval between the outcome and the predictors (9/15, 60%) [20,24-30,32] and the lack of clarity on the outcome definition (7/15, 47%) [20-22,24,26,28,30].

The third domain with risk of bias was the participants domain. A high risk of bias in the participants domain was found because inclusion and exclusion criteria were not appropriate in 2 studies (2/15, 13%) as both studies excluded participants at high risk of the outcome [27,32]. Another reason for the high risk of bias was a nonappropriate data source that was used in 1 study [20] because the authors described the study as a case-control study although the study was not nested as recommended in the PROBAST guidelines [69,70]. The predictors domain was the domain with the lowest risk of bias. The lack of clarity that resulted in an unclear risk of bias covered mainly insufficient information on whether the predictors were defined and assessed in a similar way for all participants (4/15, 27%) [18,20,22,30].

### Applicability

Overall, we found an unclear concern for applicability in 10 papers (10/15, 67%) and a low concern for applicability in 5 papers (5/15, 33%; Table 3). The unclear concern for applicability to our research question was mainly noticed in the outcome domain due to a lack of clarity in reporting the time interval between the outcomes and predictors (8/15, 53%) [20,25-30,32].

**Table 3.** Applicability per domain using the Prediction model Risk Of Bias Assessment Tool.

	Participants	Predictors	Outcome	Overall
Barnes et al [23]	Low	Low	Low	Low
Briggs et al [22]	Low	Unclear	Unclear	Unclear
Dhanda et al [32]	Low	Low	Unclear	Unclear
Dros et al [24]	Low	Low	Low	Low
Ellertsson et al [18]	Low	Unclear	Low	Unclear
Ford et al [19]	Low	Low	Low	Low
Jammeh et al [20]	Low	Unclear	Unclear	Unclear
Kocks et al [31]	Low	Low	Low	Low
LaFreniere et al [25]	Unclear	Low	Unclear	Unclear
Lin et al [26]	Unclear	Low	Unclear	Unclear
Mariani et al [21]	Low	Low	Low	Low
Nemlander et al [27]	Low	Low	Unclear	Unclear
Perveen et al [28]	Low	Unclear	Unclear	Unclear
Singh et al [29]	Low	Low	Unclear	Unclear
Su et al [30]	Unclear	Unclear	Unclear	Unclear

In the predictors domain we also found an unclear concern for applicability due to the lack of clarity in the definition of the included predictors (5/15, 33%) [18,20,22,28,30]. For example, 1 paper lacked information on how notes were annotated before they were used as predictors in the model [18]. The unclear concern for applicability in the participants' domain was mainly due to lack of information on inclusion and exclusion criteria (3/15, 20%) [25,26,30].

## Discussion

### Principal Results

We systematically reviewed the literature for studies about AI-based diagnostic prediction models for PC. These models were developed with different data sources, such as questionnaire data, secondary care data, or EHR data. Only 15 out of 49 models were developed using data from EHRs. Most of the models using EHR data focused on just 1 chronic condition. Merely 2 papers tested the model in a PC setting. All of the included studies performed at least 1 supervised AI technique, most often with random forest or logistic regression. Evaluation with the PROBAST guidelines showed an unclear to high risk of bias for all EHR papers. In most of the papers, we found unclear concerns about the applicability to our research question.

### Comparison With Prior Work

To the best of our knowledge, only 2 reviews evaluated the risk of bias in clinical prediction models on a wide range of diseases in PC studies [15,16]. Most of the included studies in these reviews showed a high to unclear risk of bias, which is in line with our findings [15,16]. However, there appear to be differences in grading compared with Abdulazeem et al [16]. They considered incomplete reporting and the absence of external validation a high risk of bias, whereas in our systematic review, these points were considered as an unclear risk of bias and no risk of bias, respectively. The study by Abbasgholizadeh et al [15] did not report details on the reasons they coded subdomains as high or unclear risk of bias, for which reason we are unable to make a formal comparison with our results.

Systematic reviews evaluating AI-based clinical prediction models in other medical fields have followed the same grading criteria as we did and found similar flaws in the analysis domain as we did in our systematic review [71,72]. These similarities include the unjustified small sample size in EHR studies, inappropriate evaluation in the performance measures, and flaws in handling of missing data [71-73].

The most used AI techniques were random forest, logistic regression, support vector machines, boosting algorithms, and neural networks. In previous systematic reviews, random forest and support vector machines are also more often found as most used methodology [16,71,73-75]. This might be explained by the well-described strong performance and ease of interpretability of random forests and support vector machines, particularly when working with lower-quality structured data. Most PC EHRs are primarily used for clinical purposes, with secondary purposes for research [69]. Thus, the challenges associated with using such EHRs to develop prediction models have been widely documented and include missing values and inconsistencies in data entry [13]. These challenges are inherent to the data and should be addressed at the preprocessing stage. We did not find papers that used generative AI methods (such as large language models). Our retrieved papers developed or validated tools based only on structured data (numbers or codes such as laboratory results, vital signs, and diagnosis codes from International Classification of Primary Care or *ICD-10* [*International Statistical Classification of Diseases, Tenth Revision*]) rather than unstructured data or written text, where large language models work well on. Literature found it valuable for the performance of the model to use unstructured data together with structured data for prognostic prediction models [76,77]. We think that future studies about diagnostic prediction tools will increasingly use generative AI methods, although it is still difficult to integrate them into clinical workflows [78].

In general, studies analyzing EHRs are subject to a high risk of bias, because these data are collected for clinical rather than research purposes [69]. Hence, clinical prediction models developed on EHRs are more difficult to reproduce and generalize, given the heterogeneity of coding systems and database infrastructures [16]. In line with models analyzed in previous studies [15,16,73], most of the clinical prediction models were not externally validated. Most of the studies developed in PC were performed in high-income countries and may not have taken into account regional or global differences in the availability of certain predictors [14-16]. For example, some predictors may not be easy to obtain in PC settings in low-income countries (eg, spirometry results for the prediction of asthma or COPD). Furthermore, the lack of stratified analyses in most studies implies that we cannot draw conclusions about how diagnostic models perform across different equity groups. Together, all these factors limit the generalizability of the clinical prediction models.

### Strengths and Limitations

The main strength of the study is the extensive search strategy with no date limit in a large and diverse range of studies on AI prediction models in PC. Not including “EHR” in the search strategy added rigor to our study as a recent review suggests that important papers could have been missed when we included EHRs in the search strategy [13]. A second strength is that the findings on the risk of bias were carefully assessed by 2 independent reviewers (LH and AC) with experience in clinical PC, and the conflicts were discussed with other experts in the field of PC and AI. Unlike previous systematic reviews that found a high proportion of studies with a high concern of applicability to the research question [72], we noticed no high concern for applicability in any study. We believe that the findings shared in our review are highly reliable in highlighting the current situation of AI studies in PC using EHRs.

The main limitation of this study is the broad definition of the terminology for the search strategy, which may have prevented us from capturing all relevant studies. For example, we included all studies that used ML and deep learning techniques. Given the lack of a widely accepted definition of AI, other reviews use other criteria for AI or ML [71,73,75]. Similarly, given our definition of diagnostic prediction models, we considered a diagnostic prediction model to be a model that predicts a health condition during a GP’s consultation. As a result, multiple prediction models that identified a disease in a database were excluded. The second limitation is the use of the PROBAST guidelines to determine the risk of bias and applicability in evaluating AI prediction models. Although the PROBAST guidelines are highly detailed and reliable in evaluating clinical prediction models [33], PROBAST has been criticized for being less specific and less applicable for AI-based models than traditional statistical methods. Considering this criticism, a protocol on the extension of PROBAST into PROBAST-Artificial Intelligence (PROBAST-AI) has been published with the aim to develop a PROBAST-AI tool to better support evaluation of prediction model studies that applied AI [3]. The PROBAST-AI tool has not yet been published.

### Future Research and Practical Implications

The relevance of the applicability of prediction models in clinical practice should be the priority when developing clinical prediction models, as stated in a number of standardized frameworks designed for prediction model developers [79,80]. We found that only 2 models were tested in PC settings. Moreover, most studies included in this review predict chronic conditions. This is also seen in previous reviews evaluating clinical prediction models in PC [14,16]. However, in general, chronic conditions are not known to be difficult to diagnose in PC. Two examples from our included papers are the diagnosis of hypertension predicted on the variable high blood pressure [25] and the diagnosis of diabetes predicted on the variable high glucose levels [28]. These predictions might not be as useful in clinical practice, even if the model performance metrics are excellent. Nevertheless, chronic conditions are highly prevalent in PC and for conditions that are influenced by several and complex factors, prediction models may facilitate the diagnostic process for the GP. As most tools focused on predicting 1 condition, GPs would have to use many prediction tools side by side to predict the correct diagnosis in daily practice. All these findings highlight that involving more practicing GPs and asking what they need are important in developing clinical prediction models with a higher success rate of clinical implementation. We recommend involving relevant stakeholders in the early stages of the development of a new model.

To improve the methodology in future studies, our findings suggest that a special focus is required on reporting areas such as methods for internal validation, appropriate inclusion of participants, and a proper sample size calculation. A high risk of bias mainly found in the analysis and outcome domains should be alarming as this questions the methodology of the included papers. We found an unclear risk of bias and unclear concern for applicability in more than half of the included studies, mainly related to poor reporting, for example, about missing data. Missing data is known as a large challenge for EHR data [13], and extra attention should therefore be paid to reporting this. Researchers can benefit from the use of the TRIPOD (Transparent Reporting of a Multivariable Prediction Model for Individual Prognosis or Diagnosis) statement [81] and PROBAST guidelines in communicating their findings [3], particularly now that the TRIPOD-AI extension is released [82]. To enhance the applicability of the prediction model, we highlight the importance of clear reporting on the time interval between predictors and outcome, a clear definition of the outcome and predictors, and a clear description of the inclusion and exclusion criteria. Differences in recording between EHRs might lower the performance of the model in the external validation step, and external validation is a crucial step for generalizable and reliable models [76]. However, we found only 2 papers that performed external validation.

### Conclusions

AI-based prediction models using EHR data are not yet ready for implementation into PC daily practice. The number of studies found was limited, and reproducibility and generalizability were insufficient. For a diagnostic prediction model to be used in PC, it is important that GPs and relevant stakeholders are involved in the development, that the model is externally validated, and that it is appropriately recorded.

## Supplementary material

10.2196/62862 Multimedia Appendix 1 Search strategy, screening guidance, table with all included papers, PROBAST (Prediction Model Risk of Bias Assessment Tool) checklist, and references of appendix.

10.2196/62862 Checklist 1 PRISMA (Preferred Reporting Items for Systematic Reviews and Meta-Analyses) 2020 checklist.

## Abbreviations

AI: artificial intelligence
CHARMS: Checklist for Critical Appraisal and Data Extraction for Systematic Reviews of Prediction Modelling Studies
COPD: chronic obstructive pulmonary disease
EHR: electronic health record
GP: general practitioner
*ICD-10*: *International Statistical Classification of Diseases, Tenth Revision*
ML: machine learning
PC: primary care
PRISMA: Preferred Reporting Items for Systematic Reviews and Meta-Analyses
PROBAST: Prediction Model Risk of Bias Assessment Tool
PROBAST-AI: Prediction Model Risk of Bias Assessment Tool-Artificial Intelligence
TRIPOD: Transparent Reporting of a Multivariable Prediction Model for Individual Prognosis or Diagnosis

## References

[1] van Smeden M, Reitsma JB, Riley RD, Collins GS, Moons KG (2021). Clinical prediction models: diagnosis versus prognosis. J Clin Epidemiol.

[2] Moons KGM, Royston P, Vergouwe Y, Grobbee DE, Altman DG (2009). Prognosis and prognostic research: what, why, and how?. BMJ.

[3] Collins GS, Dhiman P, Andaur Navarro CL (2021). Protocol for development of a reporting guideline (TRIPOD-AI) and risk of bias tool (PROBAST-AI) for diagnostic and prognostic prediction model studies based on artificial intelligence. BMJ Open.

[4] Goldstein BA, Navar AM, Carter RE (2017). Moving beyond regression techniques in cardiovascular risk prediction: applying machine learning to address analytic challenges. Eur Heart J.

[5] Liyanage H, Liaw ST, Jonnagaddala J (2019). Artificial intelligence in primary health care: perceptions, issues, and challenges. Yearb Med Inform.

[6] Mistry P (2019). Artificial intelligence in primary care. Br J Gen Pract.

[7] Summerton N, Cansdale M (2019). Artificial intelligence and diagnosis in general practice. Br J Gen Pract.

[8] Lin S (2022). A clinician’s guide to artificial intelligence (AI): Why and how primary care should lead the health care AI revolution. J Am Board Fam Med.

[9] Birks J, Bankhead C, Holt TA, Fuller A, Patnick J (2017). Evaluation of a prediction model for colorectal cancer: retrospective analysis of 2.5 million patient records. Cancer Med.

[10] Burnett B, Zhou SM, Brophy S (2023). Machine learning in colorectal cancer risk prediction from routinely collected data: a review. Diagnostics (Basel).

[11] Morgenstern JD, Buajitti E, O’Neill M (2020). Predicting population health with machine learning: a scoping review. BMJ Open.

[12] Obermeyer Z, Emanuel EJ (2016). Predicting the future—big data, machine learning, and clinical medicine. N Engl J Med.

[13] Goldstein BA, Navar AM, Pencina MJ, Ioannidis JPA (2017). Opportunities and challenges in developing risk prediction models with electronic health records data: a systematic review. J Am Med Inform Assoc.

[14] Kueper JK, Terry AL, Zwarenstein M, Lizotte DJ (2020). Artificial intelligence and primary care research: a scoping review. Ann Fam Med.

[15] Abbasgholizadeh Rahimi S, Légaré F, Sharma G (2021). Application of artificial intelligence in community-based primary health care: systematic scoping review and critical appraisal. J Med Internet Res.

[16] Abdulazeem H, Whitelaw S, Schauberger G, Klug SJ (2023). A systematic review of clinical health conditions predicted by machine learning diagnostic and prognostic models trained or validated using real-world primary health care data. PLoS One.

[17] Page MJ, McKenzie JE, Bossuyt PM (2021). The PRISMA 2020 statement: an updated guideline for reporting systematic reviews. BMJ.

[18] Ellertsson S, Loftsson H, Sigurdsson EL (2021). Artificial intelligence in the GPs office: a retrospective study on diagnostic accuracy. Scand J Prim Health Care.

[19] Ford E, Rooney P, Oliver S (2019). Identifying undetected dementia in UK primary care patients: a retrospective case-control study comparing machine-learning and standard epidemiological approaches. BMC Med Inform Decis Mak.

[20] Jammeh EA, Carroll CB, Pearson SW (2018). Machine-learning based identification of undiagnosed dementia in primary care: a feasibility study. BJGP Open.

[R21] Mariani S, Metting E, Lahr MMH, Vargiu E, Zambonelli F (2021). Developing an ML pipeline for asthma and COPD: the case of a Dutch primary care service. Int J of Intelligent Sys.

[R22] Briggs E, de Kamps M, Hamilton W, Johnson O, McInerney CD, Neal RD (2022). Machine learning for risk prediction of oesophago-gastric cancer in primary care: comparison with existing risk-assessment tools. Cancers (Basel).

[R23] Barnes DE, Zhou J, Walker RL (2020). Development and validation of eRADAR: a tool using EHR data to detect unrecognized dementia. J Am Geriatr Soc.

[R24] Dros JT, Bos I, Bennis FC (2022). Detection of primary Sjögren’s syndrome in primary care: developing a classification model with the use of routine healthcare data and machine learning. BMC Prim Care.

[R25] LaFreniere D, Zulkernine F, Barber D, Martin K Using machine learning to predict hypertension from a clinical dataset.

[R26] Lin X, Lei Y, Chen J (2023). A case-finding clinical decision support system to identify subjects with chronic obstructive pulmonary disease based on public health data. Tsinghua Sci Technol.

[R27] Nemlander E, Ewing M, Abedi E (2023). A machine learning tool for identifying non-metastatic colorectal cancer in primary care. Eur J Cancer.

[R28] Perveen S, Shahbaz M, Guergachi A, Keshavjee K (2016). Performance analysis of data mining classification techniques to predict diabetes. Procedia Comput Sci.

[R29] Singh K, Thibodeau A, Niziol LM (2022). Development and validation of a model to predict anterior segment vision-threatening eye disease using primary care clinical notes. Cornea.

[R30] Su G, Wen J, Zhu Z (2019). An approach of integrating domain knowledge into data-driven diagnostic model. Stud Health Technol Inform.

[R31] Kocks JWH, Cao H, Holzhauer B (2023). Diagnostic performance of a machine learning algorithm (asthma/chronic obstructive pulmonary disease [COPD] differentiation classification) tool versus primary care physicians and pulmonologists in asthma, COPD, and asthma/COPD overlap. J Allergy Clin Immunol Pract.

[R32] Dhanda G, Asham M, Shanks D (2023). Adaptation and external validation of pathogenic urine culture prediction in primary care using machine learning. Ann Fam Med.

[R33] Moons KGM, Altman DG, Reitsma JB, Collins GS, Transparent Reporting of a Multivariate Prediction Model for Individual Prognosis or Development Initiative (2015). New guideline for the reporting of studies developing, validating, or updating a multivariable clinical prediction model: the TRIPOD statement. Adv Anat Pathol.

[R34] Ahmed MM, Sayed AM, El Abd D (2022). Diagnosis of coronavirus disease 2019 and the potential role of deep learning: insights from the experience of Cairo University Hospitals. J Int Med Res.

[R35] Ahmed MM, Sayed AM, Khafagy GM (2022). Accuracy of the traditional COVID-19 phone triaging system and phone triage-driven deep learning model. J Prim Care Community Health.

[36] Basta M, John Simos N, Zioga M (2023). Personalized screening and risk profiles for mild cognitive impairment via a machine learning framework: implications for general practice. Int J Med Inform.

[37] Blanes-Vidal V, Lindvig KP, Thiele M, Nadimi ES, Krag A (2022). Artificial intelligence outperforms standard blood-based scores in identifying liver fibrosis patients in primary care. Sci Rep.

[38] Braido F, Santus P, Corsico AG (2018). Chronic obstructive lung disease “expert system”: validation of a predictive tool for assisting diagnosis. Int J Chron Obstruct Pulmon Dis.

[39] Brooks GJ, Ashton RE, Pethybridge RJ (1992). DERMIS: a computer system for assisting primary-care physicians with dermatological diagnosis. Br J Dermatol.

[40] Cruz-Gutiérrez V, Posada-Zamora MA, Sánchez-López A (2016). An efficient expert system for diabetes with a Bayesian inference engine. Adv Soft Comput, Micai.

[41] Dong W, Tse TYE, Mak LI (2022). Non-laboratory-based risk assessment model for case detection of diabetes mellitus and pre-diabetes in primary care. J Diabetes Investig.

[42] Exarchos TP, Rigas G, Bibas A (2016). Mining balance disorders’ data for the development of diagnostic decision support systems. Comput Biol Med.

[43] Faris H, Habib M, Faris M, Elayan H, Alomari A (2021). An intelligent multimodal medical diagnosis system based on patients’ medical questions and structured symptoms for telemedicine. Inform Med Unlocked.

[44] Farmer N, Schilstra MJ (2012). A knowledge-based diagnostic clinical decision support system for musculoskeletal disorders of the shoulder for use in a primary care setting. Shoulder Elbow.

[45] Farmer N (2014). An update and further testing of a knowledge-based diagnostic clinical decision support system for musculoskeletal disorders of the shoulder for use in a primary care setting. J Eval Clin Pract.

[46] Grill E, Groezinger M, Feil K, Strupp M (2016). Developing and implementing diagnostic prediction models for vestibular diseases in primary care. Stud Health Technol Inform.

[47] Harabor V, Mogos R, Nechita A (2023). Machine learning approaches for the prediction of hepatitis B and C seropositivity. Int J Environ Res Public Health.

[48] Heckerling PS, Canaris GJ, Flach SD, Tape TG, Wigton RS, Gerber BS (2007). Predictors of urinary tract infection based on artificial neural networks and genetic algorithms. Int J Med Inform.

[49] Hejlesen OK, Olesen KG, Dessau R, Beltoft I, Trangeled M (2005). Decision support for diagnosis of Lyme disease. Stud Health Technol Inform.

[50] Koch Nogueira PC, Venson AH, de Carvalho MFC, Konstantyner T, Sesso R (2023). Symptoms for early diagnosis of chronic kidney disease in children—a machine learning-based score. Eur J Pediatr.

[51] Liu X, Zhang W, Zhang Q (2022). Development and validation of a machine learning-augmented algorithm for diabetes screening in community and primary care settings: a population-based study. Front Endocrinol.

[52] Maizels M, Wolfe WJ (2008). An expert system for headache diagnosis: the Computerized Headache Assessment tool (CHAT). Headache.

[53] Pasic A, Pasic L, Pasic A The artificial intelligence based diagnostic assistant—AIDA.

[54] Rahimi SA, Kolahdoozi M, Mitra A (2022). Quantum-inspired interpretable ai-empowered decision support system for detection of early-stage rheumatoid arthritis in primary care using scarce dataset. Mathematics.

[55] Razzaki S, Baker A, Perov Y, Middleton K, Baxter J, Mullarkey D (2018). A comparative study of artificial intelligence and human doctors for the purpose of triage and diagnosis. arXiv.

[56] Salmeron JL, Rahimi SA, Navali AM, Sadeghpour A (2017). Medical diagnosis of Rheumatoid Arthritis using data driven PSO–FCM with scarce datasets. Neurocomputing.

[57] Sanaeifar A, Eslami S, Ahadi M, Kahani M, Vakili Arki H (2022). DxGenerator: An improved differential diagnosis generator for primary care based on MetaMap and semantic reasoning. Methods Inf Med.

[58] Shen EX, Lord A, Doecke JD (2020). A validated risk stratification tool for detecting high-risk small bowel Crohn’s disease. Aliment Pharmacol Ther.

[59] Suárez-Araujo CP, García Báez P, Cabrera-León Y (2021). A real-time clinical decision support system, for mild cognitive impairment detection, based on a hybrid neural architecture. Comput Math Methods Med.

[60] Tsoi KKF Application of artificial intelligence on a symptom diagnostic platform for telemedicine a pilot case study. https://ieeexplore.ieee.org/xpl/mostRecentIssue.jsp?punumber=8906183.

[61] Velickovski F, Ceccaroni L, Roca J (2014). Clinical Decision Support Systems (CDSS) for preventive management of COPD patients. J Transl Med.

[62] Velu SR, Ravi V, Tabianan K (2022). Data mining in predicting liver patients using classification model. Health Technol.

[63] Xiao T, Wang C, Yang M (2023). Use of virus genotypes in machine learning diagnostic prediction models for cervical cancer in women with high-risk human papillomavirus infection. JAMA Netw Open.

[64] Yoshihara A, Yoshimura Noh J, Inoue K (2022). Prediction model of Graves’ disease in general clinical practice based on complete blood count and biochemistry profile. Endocr J.

[65] Yu C, Peng YY, Liu L, Wang X, Xiao Q (2022). Leukemia can be effectively early predicted in routine physical examination with the assistance of machine learning models. J Healthc Eng.

[66] Zardab M, Balarajah V, Banerjee A (2023). Differentiating ductal adenocarcinoma of the pancreas from benign conditions using routine health records: a prospective case-control study. Cancers (Basel).

[67] Zhang H, Yin M, Liu Q (2023). Machine and deep learning-based clinical characteristics and laboratory markers for the prediction of sarcopenia. Chin Med J.

[68] Moons KGM, de Groot JAH, Bouwmeester W (2014). Critical appraisal and data extraction for systematic reviews of prediction modelling studies: the CHARMS checklist. PLoS Med.

[69] Moons KGM, Wolff RF, Riley RD (2019). PROBAST: a tool to assess risk of bias and applicability of prediction model studies: explanation and elaboration. Ann Intern Med.

[70] Wolff RF, Moons KGM, Riley RD (2019). PROBAST: a tool to assess the risk of bias and applicability of prediction model studies. Ann Intern Med.

[71] Andaur Navarro CL, Damen JAA, Takada T (2021). Risk of bias in studies on prediction models developed using supervised machine learning techniques: systematic review. BMJ.

[72] Kareemi H, Vaillancourt C, Rosenberg H, Fournier K, Yadav K (2021). Machine learning versus usual care for diagnostic and prognostic prediction in the emergency department: a systematic review. Acad Emerg Med.

[73] Andaur Navarro CL, Damen JAA, van Smeden M (2023). Systematic review identifies the design and methodological conduct of studies on machine learning-based prediction models. J Clin Epidemiol.

[74] Rajkomar A, Dean J, Kohane I (2019). Machine learning in medicine. N Engl J Med.

[75] Christodoulou E, Ma J, Collins GS, Steyerberg EW, Verbakel JY, Van Calster B (2019). A systematic review shows no performance benefit of machine learning over logistic regression for clinical prediction models. J Clin Epidemiol.

[76] Seinen TM, Fridgeirsson EA, Ioannou S (2022). Use of unstructured text in prognostic clinical prediction models: a systematic review. J Am Med Inform Assoc.

[77] Zhang D, Yin C, Zeng J, Yuan X, Zhang P (2020). Combining structured and unstructured data for predictive models: a deep learning approach. BMC Med Inform Decis Mak.

[78] Hager P, Jungmann F, Holland R (2024). Evaluation and mitigation of the limitations of large language models in clinical decision-making. Nat Med.

[79] Liu Y, Chen PH, Krause J, Peng L (2019). How to read articles that use machine learning: users’ guides to the medical literature. JAMA.

[80] Sujan M, Smith-Frazer C, Malamateniou C (2023). Validation framework for the use of AI in healthcare: overview of the new British standard BS30440. BMJ Health Care Inform.

[81] Collins GS, Reitsma JB, Altman DG, Moons KGM (2015). Transparent Reporting of a multivariable prediction model for Individual Prognosis or Diagnosis (TRIPOD): the TRIPOD statement. J Clin Epidemiol.

[82] Collins GS, Moons KGM, Dhiman P (2024). TRIPOD+AI statement: updated guidance for reporting clinical prediction models that use regression or machine learning methods. BMJ.

