# Supplementary material for: Diagnostic Prediction Models for Primary Care, Based on AI and Electronic Health Records: Systematic Review
Source: JMIR Med Inform. 2025 Aug 22;13:e62862. doi: 10.2196/62862 (PMC12373303; doi:10.2196/62862)
Supplement: Multimedia Appendix 1 [file medinform-v13-e62862-s001.docx]

Multimedia Appendix

## Search strategy

MEDLINE (4495 records retrieved on 28-08-2023)

| Primary care | Primary Health Care/ or Physicians, Family/ or Physicians, Primary Care/ or General Practitioners/ or exp General Practice/ or Community Medicine/ or Nurse Practitioners/ or Family Nurse Practitioners/ or Primary Care Nursing/ or Nurses, Community Health/ or Ambulatory Care/ or (Primary Care or Primary Health Care or Primary Healthcare or Primary Medical Care or Family Medicine or Family Healthcare or Family Health Care or Family Physician* or Family Pract* or General Practitioner* or General Practice* or Nurse Practitioner* or Family Doctor* or Family Nurse* or Community Medicine or Community Pract* or Ambulatory Care).ti,ab,kf. |
| --- | --- |
| Artificial intelligence | exp Artificial Intelligence/ or Data Mining/ or exp Decision Making, Computer Assisted/ or exp Decision Support Techniques/ or (Artificial Intelligence or Computer Heuristics or Expert System* or Fuzzy Logic or Machine Learning or Support Vector Machine or Natural Language Processing or Neural Network* or Robotic* or Deep Learning or Knowledge Representation or Automated Reasoning or Computer Vision or Data Mining or Bayesian Network* or Bayes Network* or Computer assisted diagnos* or Computer assisted therap* or Decision Support Technique* or Supervised learning or Unsupervised learning or Computer decision support).ti,ab,kf |

EMBASE (3290 records retrieved on 28-08-2023)

| Primary care | exp Primary Health Care/ or Family Medicine/ or Community Medicine/ or Family Health/ or General Practitioner/ or General Practice/ or Ambulatory Care/ or Ambulatory Care Nursing/ or Nurse Practitioner/ or Family Nurse Practitioner/ or (Primary Care or Primary Health Care or Primary Healthcare or Primary Medical Care or Family Medicine or Family Healthcare or Family Health Care or Family Physician* or Family Pract* or General Practitioner* or General Practice* or Nurse Practitioner* or Family Doctor* or Family Nurse* or Community Medicine or Community Pract* or Ambulatory Care).ti,ab,kf |
| --- | --- |
| Artificial intelligence | exp Artificial Intelligence/ or Expert System/ or Fuzzy Logic/ or exp Machine Learning/ or Natural Language Processing/ or Robotics/ or Computer Assisted Diagnosis/ or exp Computer Assisted Therapy/ or Knowledge Base/ or Ontology Development/ or (Artificial Intelligence or Computer Heuristics or Expert System* or Fuzzy Logic or Machine Learning or Support Vector Machine or Natural Language Processing or Neural Network* or Robotic* or Deep Learning or Knowledge Representation or Automated Reasoning or Computer Vision or Data Mining or Bayesian Network* or Bayes Network* or Computer assisted diagnos* or Computer assisted therap* or Decision Support Technique* or Supervised learning or Unsupervised learning or Computer decision support).ti,ab,kf. |

Web of Science (2167 records retrieved on 28-08-2023)

| Primary care | TS=(“Primary Care” OR “Primary Health care” OR “Primary Healthcare” OR “Primary Medical Care” OR “Family Medicine” OR “Family Healthcare” OR “Family Health Care” OR “Family Physician*” Or “Family Pract*” OR “General practitioner*” OR “General Practice*” OR “Nurse practitioner*” OR “Family Doctor*” OR “Family Nurse*” OR “Community Medicine” OR “Community Pract*” OR “Community Health Nurse*s” OR “Ambulatory Care”) |
| --- | --- |
| Artificial intelligence | TS=(“Artificial Intelligence” OR “Computer Heuristics*” OR “Expert System*” OR “Fuzzy Logic” OR “Machine Learning” OR “Support Vector Machine” OR “Natural Language Processing” OR “Neural Network*” OR Robotic* OR “Deep Learning” OR “Knowledge Representation” OR “Automated Reasoning” OR “Computer Vision” OR “Data Mining” OR “Bayesian Network*” OR “Bayes Network*” OR “Computer assisted diagnos*” OR “Computer assisted therap*” OR “Decision Support Technique*” OR “Supervised learning” OR “Unsupervised learning” OR “Computer decision support”) |

Cochrane (705 records retrieved on 28-08-2023)

| Primary care | [mh ^"Primary Health Care"] OR [mh ^"Physicians, Family"] OR [mh ^"Physicians, Primary Care"] OR [mh ^"General Practitioners"] OR [mh "General Practice"] OR [mh ^"Community Medicine"] OR [mh ^"Nurse Practitioners"] OR [mh ^"Family Nurse Practitioners"] OR [mh ^"Primary Care Nursing"] OR [mh ^"Nurses, Community Health"] OR [mh ^"Ambulatory Care"]  OR ("Primary Care":ti,ab,kw OR "Primary Health Care":ti,ab,kw OR "Primary Healthcare":ti,ab,kw OR "Primary Medical Care":ti,ab,kw OR "Family Medicine":ti,ab,kw OR "Family Healthcare":ti,ab,kw OR "Family Health Care":ti,ab,kw OR ("Family" NEXT Physician*):ti,ab,kw OR ("Family" NEXT Pract*):ti,ab,kw OR ("General" NEXT Practitioner*):ti,ab,kw OR ("Nurse" NEXT Practitioner*):ti,ab,kw OR ("Family" NEXT Doctor*):ti,ab,kw OR ("Family" NEXT Nurse*):ti,ab,kw OR "Community Medicine":ti,ab,kw OR ("Community" NEXT Pract*):ti,ab,kw OR "Ambulatory Care":ti,ab,kw) |
| --- | --- |
| Artificial intelligence | [mh "Artificial Intelligence"] OR [mh ^"Data Mining"] OR [mh "Decision Making, Computer Assisted"] OR [mh "Decision Support Techniques"] OR ("Artificial Intelligence":ti,ab,kw OR "Computer Heuristics":ti,ab,kw OR ("Expert" NEXT System*):ti,ab,kw OR "Fuzzy Logic":ti,ab,kw OR "Machine Learning":ti,ab,kw  OR "Support Vector Machine":ti,ab,kw OR "Natural Language Processing":ti,ab,kw OR ("Neural" NEXT Network*):ti,ab,kw OR Robotic*:ti,ab,kw OR "Deep Learning":ti,ab,kw OR "Knowledge Representation":ti,ab,kw OR "Automated Reasoning":ti,ab,kw OR "Computer Vision":ti,ab,kw OR "Data Mining":ti,ab,kw OR "Supervised learning":ti,ab,kw  OR "Unsupervised learning":ti,ab,kw OR ("Bayesian" NEXT Network*):ti,ab,kw  OR ("Bayes" NEXT Network*):ti,ab,kw) OR ("Decision Support" NEXT Technique*):ti,ab,kw OR"Computer decision support":ti,ab,kw |

## Screening guidance

Last updated 22-12-2023

### Screening guidance title/abstract screening

**Exclusion list in for title/abstract screening in Rayyan**

Excluding based on order of numbers, for example if papers are excluded because they have no primary care focus, and it is not about a diagnostic prediction tool, excluding for reason 1 suffices.

Reasons for exclusion should be added per reviewer manually. Number them as follows:

1. No primary Care (PC) focus;
2. No artificial intelligence. See II. For more information. If technique is not stated clearly, include for full text screening;
3. No diagnostic prediction tool. If diagnostic or prognostic is not clear, include for full text screening;
4. No use of existing database with primary care data. See III. and IV. For more information. If data seems from primary care but some variables are secondary care, include for full text screening (difference between primary and secondary care differs between countries);
5. Wrong study type. Not an observational or intervention study, but e.g. systematic review, meta-analyses, case study, editorial;
6. Not written in English or Dutch language;
7. No abstract available.
8. Conference abstract

**Inclusion criteria general**

We included all studies about the development of diagnostic prediction tools in primary care. We restricted our inclusion to tools developed with artificial intelligence. To be included, the study had to meet the following inclusion criteria:

I. The prediction tool has been developed with artificial intelligence. Examples are:

- To identify patterns and risks
  - Example: a study where a statistical machine learning model is used to develop a new risk prediction tool for CVD in primary care data, should be INCLUDED.
  - A few key machine learning methods and terms to look for include (but are not limited to): ‘random forests’, ‘neural networks’, ‘ensemble methods’, ‘supervised, unsupervised or semi-supervised models’.
- Studies including (methods for) natural language processing (e.g. deriving relevant text from clinical notes and/or converting such relevant text to numerical variables or otherwise research-usable data)
- Studies describing the development or evaluation of practical applications or software (e.g. different/novel use or improvement of already existing tools in general practice)

II. The prediction tool is diagnostic, not prognostic. Prediction models are developed to aid health care providers in estimating the probability that a specific disease or condition is present (diagnostic models) [33]. We focus in this paper on new diagnosis. If the timing of the predicted outcome was unclear, the study was included for full text screening.

- Types of studies we defined as not diagnostic
  - For example predicting suicide risk
  - For example papers about frailty or falling.
- Exclude studies that don’t predict the risk of a disease at this moment, but for the future.
- A tool that predicts an event within a few months before onset of a particular disease.
  - For example a tool designed to prescreen a group of people at risk for specific diseases.
- Exclude tools that aren’t for patients
  - For example a prediction tool to predict the amount of influenza patients in a sample.
  - For example a tool that identifies overlooked cases or groups or clusters or patients at risk.
- Exclude tools that don’t focus on new diagnosis
  - For example asthma exacerbations for patients with asthma.

III. The prediction tool is developed for primary care. The direct relevance to primary care should be clear, for instance:

- The aim of the study is to generate results of specific relevance for use in primary care.
  - Studies using (partly) secondary care data such as hospital data and/or discharge notes, or studies involving a patient population being discharged from hospital, need to specify in the abstract that the aim/methods/results are relevant for use in primary care settings in order to be included.
- The study is conducted in primary care or with primary care data.
- Exclude studies performed in secondary care
- Exclude papers about dental care

List of diagnostic tests or diagnostic test data that can or can’t be considered as primary care:

Consider as primary care:

- X-rays, for example chest x-rays or x-rays to detect fractures
- Ultrasounds, most types
- Lab results
- Heart or lung sounds that can normally be performed using a stethoscope
- Retina screening (fundoscopy), can be performed in some GP practices, therefore include
- ECG (Electrocardiography)

Don’t consider this as primary care:

- MRI + CT
- Invasive diagnostics, such as colonoscopy, endoscopy, arthroscopy
- Genetic diagnostic tests
- EEG (Electroencephalography)
- Echocardiography
- Optical Coherence Tomography

IV**.** The study is an observational or trial/intervention study

- We will exclude reviews, meta-analyses, case studies, editorials, animal studies.
- We will exclude study protocols

V. The article has been written in English or Dutch language.

VI. Abstract and full text should be available.

### Screening guidance full text screening

Last updated 22-12-2023

II. The prediction tool is diagnostic

- - Diagnostic tests instead of diagnostic prediction tools, meant for additional investigation or diagnostic tests should be excluded:
  - For example Gamlyn, 1999 - The development of a neural network-based ambulatory ECG monitor. This paper describes the development of an ECG monitor that doesn’t provide the medical diagnosis.
  - For example Chang, 2021 - Computational Methods to Measure Patterns of Gaze in Toddlers with Autism Spectrum Disorder. This tool detects symptoms and doesn’t provide the physician with a diagnosis.

Diagnostic tests that predict results of physical examination and not diagnoses should be excluded

- For example Lang, 2020 - Graph-based semi-supervised one class support vector machine for detecting abnormal lung sounds. They detect lung sounds without predicting a diagnosis.
- Prediction tools that are not able to predict a diagnosis at the moment of consultation should be excluded
  - For example Akyea, 2020 – Performance and clinical utility of supervised machine-learning approaches in detecting familial hypercholesterolemia in primary care. They detect cases and not predict the diagnosis in a consultation.
  - For example Kop, 2016 – Predictive modeling of colorectal cancer using a dedicated pre-processing pipeline on routine electronic medical records. They create a pipeline to use EHR for predictions, they do not predict a diagnosis
- That doesn’t predict a diagnosis, but predicts a subtype within a disease
  - For example Kaneko 2021 - Machine learning based models for prediction of subtype diagnosis of primary aldosteronism using blood test. They predict subtypes of primary aldosteronism.

III. The prediction tool is developed for primary care

- If primary care is not mentioned in the aim, the tool should either be developed with primary care data or tested on/in primary care. If not, the paper should be excluded.
- For example Perveen, 2016 - Performance Analysis of Data Mining Classification Techniques to Predict Diabetes. This paper doesn’t describe PC in the aim but is tested on a PC dataset.

We excluded papers data based on imaging. Or where images where used to get to the diagnosis, for example about skin lesions.

- For example Escale-Besa, 2023 - Exploring the potential of artificial intelligence in improving skin lesion diagnosis in primary care. This tool is based on skin images.
- For example Liu 2022 - depression based on fascial expression: integrated facial activities and gaze data to establish an automatic recognition model of Center for Epidemiologic Studies Depression Scale scores. This tool is based on facial imaging.

Heart and lung sounds were excluded.

- For example Shokouhm, 2023: Diagnosis of Coexisting Valvular Heart Diseases Using Image-to-Sequence Translation of Contact Microphone Recordings.

IV. The data source for the prediction tool is electronic health records (EHR). For the selection of papers about EHR, we considered papers that included (routine) registration data or clinical notes from the general practitioner. This had to be stated in the paper.

- Only including information about medical history without stating where the data comes from is not included, because this can also be derived from interviewing or questionnaires.
  - For example Dong, 2022 - Non-laboratory-based risk assessment model for case detection of diabetes mellitus and pre-diabetes in primary care. They are not clear where there data comes from.

## All extracted papers

Basic information from non-EHR papers.

| **Author, year** | **Title** | **Country*** | **Study design** | **Outcome** | **Data source** |
| --- | --- | --- | --- | --- | --- |
| Ahmed et al (2022) [34] | Diagnosis of coronavirus disease 2019 and the potential role of deep learning: insights from the experience of Cairo University Hospitals | Egypt | Retrospective | COVID-19 | Datasets from primary care, EHR not mentioned. Secondary care data |
| Ahmed et al (2022) [35] | Accuracy of the Traditional COVID-19 Phone Triaging System and Phone Triage-Driven Deep Learning Model | Egypt | Retrospective | COVID-19 | Datasets from primary care, EHR not mentioned. Secondary care data |
| Basta et al (2023) [36] | Personalized screening and risk profiles for Mild Cognitive Impairment via a Machine Learning Framework: Implications for general practice | Greece | Retrospective | Mild Cognitive Impairment | Questionnaire data |
| Blanes-Vidal et al (2022) [37] | Artificial intelligence outperforms standard blood-based scores in identifying liver fibrosis patients in primary care | Denmark | Prospective | Clinically significant liver stiffness(fibrosis) | Population data from recruited participants |
| Braido et al (2018) [38] | Chronic obstructive lung disease "expert system": Validation of a predictive tool for assisting diagnosis | Italy | Retrospective | Chronic obstructive lung disease | Specialists’ knowledge |
| Brooks et al (1992) [39] | DERMIS: a computer system for assisting primary-care physicians with dermatological diagnosis | UK | Prospective | A differential diagnosis for skin diseases | Secondary care patients |
| Cruz-Gutierrez et al (2016) [40] | An Efficient Expert System for Diabetes with a Bayesian Inference Engine | Mexico | Retrospective | Diabetes Mellitus 2 | Specialists’ knowledge |
| Dong et al (2022) [41] | Non-laboratory-based risk assessment model for case detection of diabetes mellitus and pre-diabetes in primary care | China/Hong Kong | Retrospective | Undiagnosed diabetes mellitus and pre-diabetes mellitus | Questionnaire data |
| Exarchos et al (2016) [42] | Mining balance disorders' data for the development of diagnostic decision support systems | Greece, UK, Belgium, Germany | Retrospective | diagnosis of balance disorders | Secondary care data |
| Faris et al (2021) [43] | An intelligent multimodal medical diagnosis system based on patients' medical questions and structured symptoms for telemedicine | Data from Middle East and North Africa | Retrospective | Symptom checker for multiple symptoms | Consultations data from telemedicine company, primary care EHR data unknown |
| Farmer and Schilstra (2012) [44] | A Knowledge-based Diagnostic Clinical Decision Support System for Musculoskeletal Disorders of the Shoulder for Use in a Primary Care Setting | UK | Retrospective | Musculoskeletal shoulder disorders | Primary care clinical audit data |
| Farmer (2014) [45] | An update and further testing of a knowledge-based diagnostic clinical decision support system for musculoskeletal disorders of the shoulder for use in a primary care setting | UK | Prospective | Musculoskeletal shoulder complaints | Secondary care data |
| Grill et al(2016) [46] | Developing and Implementing Diagnostic Prediction Models for Vestibular Diseases in Primary Care | Germany | Retrospective | Vestibular disease | Secondary care data |
| Harabor et al (2023) [47] | Machine Learning Approaches for the Prediction of Hepatitis B and C Seropositivity | Romania | Prospective | Hepatitis B and C seropositivity | Questionnaire data |
| Heckerling et al (2007) [48] | Predictors of urinary tract infection based on artificial neural networks and genetic algorithms | USA | Retrospective | Urinary tract infection | Primary care data from recruited patients |
| Hejlesen et al (2005) [49] | Decision support for diagnosis of Lyme disease | Denmark | Retrospective | Lyme disease | Unclear data source |
| Koch Nogueira et al (2023) [50] | Symptoms for early diagnosis of chronic kidney disease in children - a machine learning-based score | Brazil | Retrospective | Chronic kidney disease in children | Secondary care data |
| Liu et al (2022) [51] | Development and validation of a machine learning-augmented algorithm for diabetes screening in community and primary care settings: A population-based study | China | Retrospective | Diabetes | Questionnaire data |
| Maizels and Wolfe (2008) [52] | An expert system for headache diagnosis: The computerized headache assessment tool (CHAT) | USA | Retrospective | Headaches | Medical guidelines |
| Pasic et al (2022) [53] | The Artificial Intelligence Based Diagnostic Assistant - AIDA | Open source data. Authors from Hungary | Retrospective | Multiple diagnoses | Open source dataset, no primary or secondary care data |
| Rahimi et al (2022) [54] | Quantum-Inspired Interpretable AI-Empowered Decision Support System for Detection of Early-Stage Rheumatoid Arthritis in Primary Care Using Scarce Dataset | Iran | Retrospective | Rheumatoid Arthritis | Secondary care data |
| Razzaki et al (2018) [55] | A comparative study of artificial intelligence and human doctors for the purpose of triage and diagnosis | UK | Prospective | Multiple diagnoses | Epidemiological data, unclear data and specialists’ knowledge |
| Salmeron et al (2017) [56] | Medical diagnosis of Rheumatoid Arthritis using data driven PSO-FCM with scarce datasets | Experts from Canada. Authors from: Spain, Chile, Czech Republic | Retrospective | Rheumatoid Arthritis | Specialists’ knowledge |
| Sanaeifar et al (2022) [57] | DxGenerator: An Improved Differential Diagnosis Generator for Primary Care Based on MetaMap and Semantic Reasoning | Open source data. Authors from Iran | Retrospective | To design and evaluate a novel practical web-based differential diagnosis generator solution in primary care (for gastrointestinal complications that cause abdominal pain) | Specialists’ knowledge |
| Shen et al (2020) [58] | A validated risk stratification tool for detecting high-risk small bowel Crohn's disease | Australia | Retrospective + prospective | Ileal Chron’s disease | Secondary care data |
| Suarez-Araujo et al (2021) [59] | A Real-Time Clinical Decision Support System, for Mild Cognitive Impairment Detection, Based on a Hybrid Neural Architecture | USA | Retrospective | Mild Cognitive Impairment | Secondary care data |
| Tsoi (2019) [60] | Application of Artificial Intelligence on a Symptom Diagnostic Platform for Telemedicine A Pilot Case Study | China | Retrospective | Multiple diagnoses | Primary and secondary care data from recruited patients |
| Velickovski et al (2014) [61] | Clinical Decision Support Systems (CDSS) for preventive management of COPD patients | Spain | Retrospective | COPD | Secondary care data |
| Velu et al (2022) [62] | Data mining in predicting liver patients using classification model | Open source data. Authors from: Saudi Arabia and Malaysia | Retrospective | Liver patients | Secondary care data |
| Xiao et al (2023) [63] | Use of Virus Genotypes in Machine Learning Diagnostic Prediction Models for Cervical Cancer in Women with High-Risk Human Papillomavirus Infection | China | Retrospective | High-Risk Human Papillomavirus Infection | Primary care data, no EHR. Patients from screening program. |
| Yoshihara et al (2022) [64] | Prediction model of Graves' disease in general clinical practice based on complete blood count and biochemistry profile | Japan | Retrospective | Graves’ disease and thyrotoxicosis | Secondary care data |
| Yu et al (2022) [65] | Leukemia can be Effectively Early Predicted in Routine Physical Examination with the Assistance of Machine Learning Models | China | Retrospective | Leukemia | Secondary care data |
| Zardab et al (2023) [66] | Differentiating Ductal Adenocarcinoma of the Pancreas from Benign Conditions Using Routine Health Records: A Prospective Case-Control Study | UK | Prospective | Pancreatic ductal adeno-carcinoma | Secondary care data |
| Zhang et al (2023) [67] | Machine and deep learning-based clinical characteristics and laboratory markers for the prediction of sarcopenia | China | Retrospective | Sarcopenia | Secondary care data |

* country of collected data to train/test/validate/externally validate the model

| PROBAST Checklist | | | | |
| --- | --- | --- | --- | --- |
| **Domain 1: participants** | | | | |
|  | 1.1 Were appropriate data sources used? | 1.2 Were all inclusions and exclusions of participants appropriate? | *Domain-level Risk of Bias judgement* | *Domain-level applicability judgement* |
| Barnes et al [23] | Yes | Yes | Low, clear inclusion and exclusion criteria | Low concerns |
| Briggs et al [22] | Yes | Yes | Low, appropriate in- and exclusion criteria, nested case-control study | Low concerns |
| Dhanda et al [32] | Yes | No | High, high risk features were excluded, with these features it is easier to diagnose. | Low concerns, only applicable to low risk population in GP practices |
| Dros et al [24] | Yes | Yes | Low | Low concerns |
| Ellertsson et al [18] | Yes | Yes | Low | Low concerns |
| Fordet al [19] | Yes | No information | Unclear | Low concerns |
| Jammeh et al [20] | No, Case control study, not nested | Yes | High, wrong study design | Low concerns |
| Kocks et al [31] | Yes | Yes | Low | Low concerns |
| LaFreniere et al [25] | No information | No information, in- and exclusion criteria unclear. | Unclear, case control study, nested unclear. In- and exclusion criteria unclear. | Unclear concerns, in- and exclusion criteria unclear. Matching unclear from case-control study |
| Lin et al [26] | Yes | No information, In- and exclusion criteria unclear | Unclear, in- and exclusion criteria unclear. | Unclear concerns, in- and exclusion criteria unclear. |
| Mariani et al [21] | Yes | No information, reason unclear for excluding patients with no follow up at 3 or 12 months | Unclear, exclusion reason unclear | Low concerns |
| Nemlander et al [27] | Yes | No, excluding patients with previous cancer. They have a higher change of getting cancer | High, exclusion criteria incorrect | Low concerns, only applicable to patients without previous cancer |
| Perveenet al [28] | Probably Yes | Probably Yes | Low | Low concerns |
| Singh et al [29] | Yes | Yes | Low | Low concerns |
| Su et al [30] | Yes | No information, in- and exclusion criteria unclear | Unclear, in- and exclusion criteria unclear. | Unclear concerns, in- and exclusion criteria unclear |

| **Domain 2 predictors** | | | | |  |
| --- | --- | --- | --- | --- | --- |
|  | 2.1 Were predictors defined and assessed in a similar way for all participants? | 2.2 Were predictor assessment made without knowledge of outcome data? | 2.3 Are all predictors available at the time the model is intended to be used? | *Domain-level Risk of Bias judgement* | *Domain-level applicability judgement* |
| Barnes et al [23] | No information | Probably Yes | Yes | Low | Low concerns, predictors could be known at time of prediction |
| Briggs et al [22] | Yes | Yes | Yes | Unclear, not clear how lab results are measured | Unclear concerns, unclear how lab results are measured, if it is the same for all PC settings |
| Dhanda et al [32] | Yes | Yes | Yes | Low | Low concerns |
| Dros et al [24] | Yes | Probably Yes | Yes | Low | Low concerns |
| Ellertsson et al [18] | No information, unclear about annotated notes | Yes | Yes | Unclear, lack of information about annotated notes | Unclear concerns because of annotated notes |
| Ford et al [19] | Yes | Probably Yes | Yes | Low | Low concerns |
| Jammeh et al [20] | No information, unclear what predictors are used and how they are defined | Probably Yes | Probably Yes | Unclear, unclear what predictors are added to the final model | Unclear concerns,  Unclear what predictors are added |
| Kocks et al [31] | Yes | Yes | Yes | Low | Low concerns |
| LaFreniere et al [25] | Yes | Probably Yes | Probably Yes | Low | Low concerns |
| Lin et al [26] | Yes | Yes | Yes | Low | Low concerns |
| Mariani et al [21] | Yes | Yes | Yes | Low | Low concerns |
| Nemlander et al [27] | Yes | Yes | Yes | Low | Low concerns |
| Perveen et al [28] | Yes | No information, no information provided | No information, no information provided | Unclear, missing information about predictors | Unclear concerns, important information not reported. Extra concern because they consider DM type 1 and type 2 as one, this should be seen as two different diagnoses |
| Singh et al [29] | Yes | Yes | Yes | Low | Low concerns |
| Su et al [30] | No information | Yes | Yes | Unclear, unclear if predictors are assessed in a similar way | Unclear concerns, list of predictors unclear |

| **Domain 3: Outcome** | | | | |  |
| --- | --- | --- | --- | --- | --- |
|  | 3.1 was the outcome determined appropriately? | 3.2 Was a prespecified or standard outcome definition used? | 3.3 Were predictors excluded from the outcome definition? | 3.4 Was the outcome defined and determined in a similar way for all participants? | 3.5 Was the outcome determine without knowledge of predictor information? |
| Barnes et al [23] | Yes | Yes | Yes | Yes | Yes |
| Briggs et al [22] | No information | No information | Yes | Probably Yes | Probably Yes |
| Dhanda et al [32] | Yes | Yes | Yes | Yes | Yes |
| Dros et al [24] | Yes | No information | Yes | Yes | Probably Yes |
| Ellertsson et al [18] | Yes | Yes | Yes | Yes | Yes |
| Ford et al [19] | Yes | Yes | Yes | Yes | Yes |
| Jammeh et al [20] | Yes | No information, no clear definition of the outcome | No information, predictors in final model unclear | Yes | Yes |
| Kocks et al [31] | Yes | Yes | No, spirometry scores (predictors) part of outcome | Probably Yes | No, spirtometry scores (predictors) part of the outcome |
| LaFreniere et al [25] | Yes | Yes | Yes | Yes | Yes |
| Lin et al [26] | No information, unclear outcome determination | No information, outcome definition unclear | Yes | Yes | Yes |
| Mariani et al [21] | Yes | No information, determination of outcome unclear | Yes | Yes | Yes |
| Nemlander et al [27] | Yes | Yes | Yes | Yes | Yes |
| Perveen et al [28] | Probably Yes | No information, outcome definition unclear | No, glucose levels (predictor) part of outcome | Probably Yes | Probably Yes |
| Singh et al [29] | Yes | Yes | Probably Yes | Yes | Yes |
| Su et al [30] | Probably Yes | No information | No information | No information | Probably Yes |

| **Domain 3: Outcome** | | | | **Domain 4: Analysis** |
| --- | --- | --- | --- | --- |
|  | 3.6 Appropriate time interval between predictor assessment and outcome? | *Domain-level Risk of Bias judgement* | *Domain-level applicability judgement* | 4.1 Were there a reasonable number of participants with the outcome? |
| Barnes et al [23] | Yes | Low | Low concerns | Yes |
| Briggs et al [22] | Yes | Unclear, Unclear how outcome is determined | Unclear concerns, lack of outcome definition | Yes |
| Dhanda et al [32] | No information, time interval not unclear | Unclear, time interval unclear | Unclear concerns, time interval unknown | Yes |
| Dros et al [24] | No information,  time interval unclear | Unclear, unclear how outcome is determined | Low concerns and not unclear, because it takes years on average to give the right diagnosis | No |
| Ellertsson et al [18] | Yes | Low | Low concerns | No |
| Ford et al [19] | Probably Yes | Low | Low concerns | Yes |
| Jammeh et al [20] | No information,  time interval unclear | Unclear, unclear reporting of outcome, predictors and time interval | Unclear concerns, timing and outcome unclear | No information, number of predictors unknown |
| Kocks et al [31] | Probably Yes | High, predictors not excluded from outcome definition | Low concerns | No, participants with outcome too low |
| LaFreniere et al [25] | No information, time interval unclear | Unclear, time interval unclear | Unclear concerns, time interval unknown | Yes |
| Lin et al [26] | No information, time interval unclear | Unclear, outcome definition and time interval unclear | Unclear concerns, time interval unknown | No, participants with outcome too low |
| Mariani et al [21] | Yes | Unclear, time interval unclear | Low concerns | No information, number of participants with outcome unclear |
| Nemlander et al [27] | No information, time interval unclear | Unclear, time interval unclear | Unclear concerns, time interval unknown | Yes |
| Perveen et al [28] | No information, time interval unclear | High, predictors not excluded from outcome definition | Unclear concerns, time interval unknown | Yes |
| Singh et al [29] | No information, time interval unclear | Unclear, time interval unclear | Unclear concerns, time interval unknown | No, participants with outcome too low |
| Su et al [230] | No information, time interval unclear | Unclear, a lot of missing information | Unclear concerns, time interval and outcome definition unclear | No information |

| **Domain 4 Analysis** | | | | | |
| --- | --- | --- | --- | --- | --- |
|  | 4.2 Were continuous and categorical predictors handled appropriately? | 4.3 Were all enrolled participants included in the analysis? | 4.4. Were participants with missing data handled appropriately? | 4.5 Was selection of predictors based on univariable analysis avoided? | 4.6 Were complexities in the data accounted for appropriately? |
| Barnes et al [23] | Yes | Yes | No information, unclear how missings are handled | Yes | Yes |
| Briggs et al [22] | Yes | Probably Yes | No information, unclear how missings are handled | Yes | Yes |
| Dhanda et al [32] | Yes | Yes | No information, unclear how missings are handled | Yes | Yes |
| Dros et al [24] | Yes | Yes | Yes | Yes | Yes |
| Ellertsson et al [18] | Probably Yes | Yes | No information, unclear how missings are handled | Probably Yes | Probably Yes |
| Ford et al [19] | Yes | Yes | No information, unclear how missings are handled | Yes | Yes |
| Jammeh et al [20] | Probably Yes | Yes | No information, unclear how missings are handled | Probably Yes | Probably Yes |
| Kocks et al [31] | Yes | Yes | Yes | *NA* | Yes |
| LaFreniere et al [25] | Probably Yes | Yes | Yes | Probably Yes | Probably Yes |
| Lin et al [26] | Yes | Yes | Yes | Yes | Yes |
| Mariani et al [21] | Yes | Yes | Yes | Yes | Probably Yes |
| Nemlander et al [27] | Yes | Yes | No information, unclear how missings are handled | Probably Yes | Probably Yes |
| Perveen et al [28] | Probably Yes | No, participants excluded, for unclear reasons | No information, unclear how missings are handled | No information | Probably Yes |
| Singh et al [29] | Probably Yes | Yes | No information, unclear how missings are handled | Yes | Probably Yes |
| Su et al [30] | No information | No information | No information, unclear how missings are handled | Yes | No information |

| **Domain 4: Analysis** | | | | | **Overall judgement** | |
| --- | --- | --- | --- | --- | --- | --- |
|  | 4.7 Were relevant model performance measures evaluated appropriately? | 4.8 Were model overfitting and optimism in model performance accounted for? | 4.9 Do predictors and their assigned weights in the final model correspond to the results from the reported multivariable analysis? | *Domain-level Risk of Bias judgement* | ***Risk of Bias*** | ***Applicability*** |
| Barnes et al [23] | Yes, assessed calibration | Yes | Yes | Unclear, unclear about handling of missing data | Unclear | Low concerns |
| Briggs et al [22] | Yes, assessed calibration | Yes | Yes | Unclear, unclear about handling of missing data | Unclear | Unclear concerns |
| Dhanda et al [32] | Yes, assessed calibration | Yes | Yes | Unclear, unclear about handling of missing data | High | Unclear concerns |
| Dros et al [24] | Yes calibration not mentioned | Yes | Yes | High,  No reasonable number of participants with the outcome | High | Low concerns |
| Ellertsson et al [18] | Yes, calibration not mentioned | Yes | No information, predictors in final model unknown | High, no reasonable number of participants with outcome | High | Unclear concerns |
| Ford et al [19] | Yes, calibration not mentioned | Probably yes | Yes | Unclear, unclear about handling of missing data | Unclear | Low concerns |
| Jammeh et al [20] | Yes, calibration not mentioned | Yes | No information, predictors in final model unknown | Unclear about missings and predictors in final model unknown | High | Unclear concerns |
| Kocks et al [31] | No, no calibration or discrimination | *Not applicable* | *Not applicable* | High, participants with outcome too low, performance metrics irrelevant | High | Low concerns |
| LaFreniere et al [25] | Yes, calibration not mentioned | Probably yes | No information, predictors in final model unknown | Unclear, predictors in final model unknown | Unclear | Unclear concerns |
| Lin et al [26] | Yes, calibration not mentioned | Yes | No information, predictors in final model unknown | High, participants with outcome too low | High | Unclear concerns |
| Mariani et al [21] | Yes, calibration not mentioned | Yes | No information, predictors in final model unknown | Unclear about missings and predictors in final model unknown | Unclear | Low concerns |
| Nemlander et al [27] | Yes, calibration not mentioned | Yes | No information, predictors in final model unknown | Unclear about missings and predictors in final model unknown | High | Unclear concerns |
| Perveen et al [28] | No Just AROC is not sufficient | No information | No information, predictors in final model unknown | High, relevant performance metrics are missing | High | Unclear concerns |
| Singh et al [29] | Yes, assessed calibration | Yes | No information, predictors in final model unknown | High,  participants with outcome too low | High | Unclear concerns |
| Su et al [30] | Yes, calibration not mentioned | Yes | No information, predictors in final model unknown | Unclear, a lot of missing information | Unclear | Unclear concerns |
